# A Novel Indication for Panobinostat as a Senolytic Drug in NSCLC and HNSCC

**DOI:** 10.1038/s41598-017-01964-1

**Published:** 2017-05-15

**Authors:** Leleesha Samaraweera, Alfred Adomako, Alicia Rodriguez-Gabin, Hayley M. McDaid

**Affiliations:** 1 Department of Molecular Pharmacology, Albert Einstein College of Medicine, Bronx, NY 10461 USA; 2 Department of Pathology, Albert Einstein College of Medicine, Bronx, NY 10461 USA; 3 Medicine (Oncology), Albert Einstein College of Medicine, Bronx, NY 10461 USA

## Abstract

Panobinostat (pano) is an FDA-approved histone deacetylase inhibitor. There is interest in evaluating alternate dosing schedules and novel combinations of pano for the treatment of upper aerodigestive and lung malignancies; thus we evaluated it in combination with Taxol, a chemotherapeutic with activity in both diseases. Dose-dependent synergy was observed in Non-Small Cell Lung Cancer (NSCLC) and Head and Neck Squamous Cell Carcinoma (HNSCC) cell lines and was due to senescence rather than potentiation of cell death. Senescence occurred following cisplatin- or Taxol-treatment in cell lines from both cancer types and was associated with decreased histone 3 (H3) acetylation and increased Bcl-xL expression: the latter a biomarker of senescence and target of anti-senescence therapeutics, or senolytics. Since H3 acetylation and Bcl-xL expression were altered in senescence, we subsequently evaluated pano as a senolytic in chemotherapy-treated cancer cells enriched for senescent cells. Pano caused cell death at significantly higher rates compared to repeat dosing with chemotherapy. This was associated with decreased expression of Bcl-xL and increased acetylated H3, reversing the expression patterns observed in senescence. These data support evaluating pano as a post-chemotherapy senolytic with the potential to kill persistent senescent cells that accumulate during standard chemotherapy in NSCLC and HNSCC.

## Introduction

Despite intense cisplain-based chemoradiotherapy^1, 2^, the majority of Non-Small Cell Lung Cancer (NSCLC) and Head and Neck Squamous Cell Carcinoma (HNSCC) patients have short disease-free survival and quickly succumb to metastatic disease; therefore there is a need to evaluate novel combinations to evolve treatment modalities that can extend survival. Although taxotere is FDA-approved for the treatment of HNSCC, the role of Taxol in the disease has not been fully evaluated in these therapeutically recalcitrant malignancies.

Histone deacetylases (HDAC) remove acetyl groups in the lysine residues of histones and non-histone proteins^3^ and thus regulate important cellular functions including gene expression, differentiation, proliferation and survival^3, 4^. Aberrant expression and mutation of HDACs have been documented in various malignancies^4^, prompting the development of therapeutic inhibitors, some of which are FDA-approved for the treatment of refractory cutaneous T-cell lymphoma, peripheral T-cell lymphoma and multiple myeloma^3, 5^. Panobinostat (pano) is a class I, II and IV HDAC inhibitor (HDACi) that is FDA-approved for the treatment of refractory Multiple myeloma^5^. HDACi’s, including pano, have also been evaluated in early phase clinical studies for the treatment of both HNSCC and NSCLC^6–9^. Despite evidence of promising anti-tumor activity, particularly in combination with other epigenetic modulators^10^, toxicity remains a therapeutic challenge^8^. Thus continued development and evaluation of novel pano combinations is required.

Here we explore the efficacy of pano in combination with Taxol in HNSCC and NSCLC, and show synergistic proliferative arrest via induction of senescence. Subsequently, we extend these studies to evaluate the efficacy of single-agent pano as a post-chemotherapy senolytic therapy that causes senescent cell death and is more efficacious than retreating cancer cells with successive cycles of chemotherapy.

## Results

### The Combination of Taxol and Pano is Synergistic in NSCLC and HNSCC Cells

**Figure 1:**
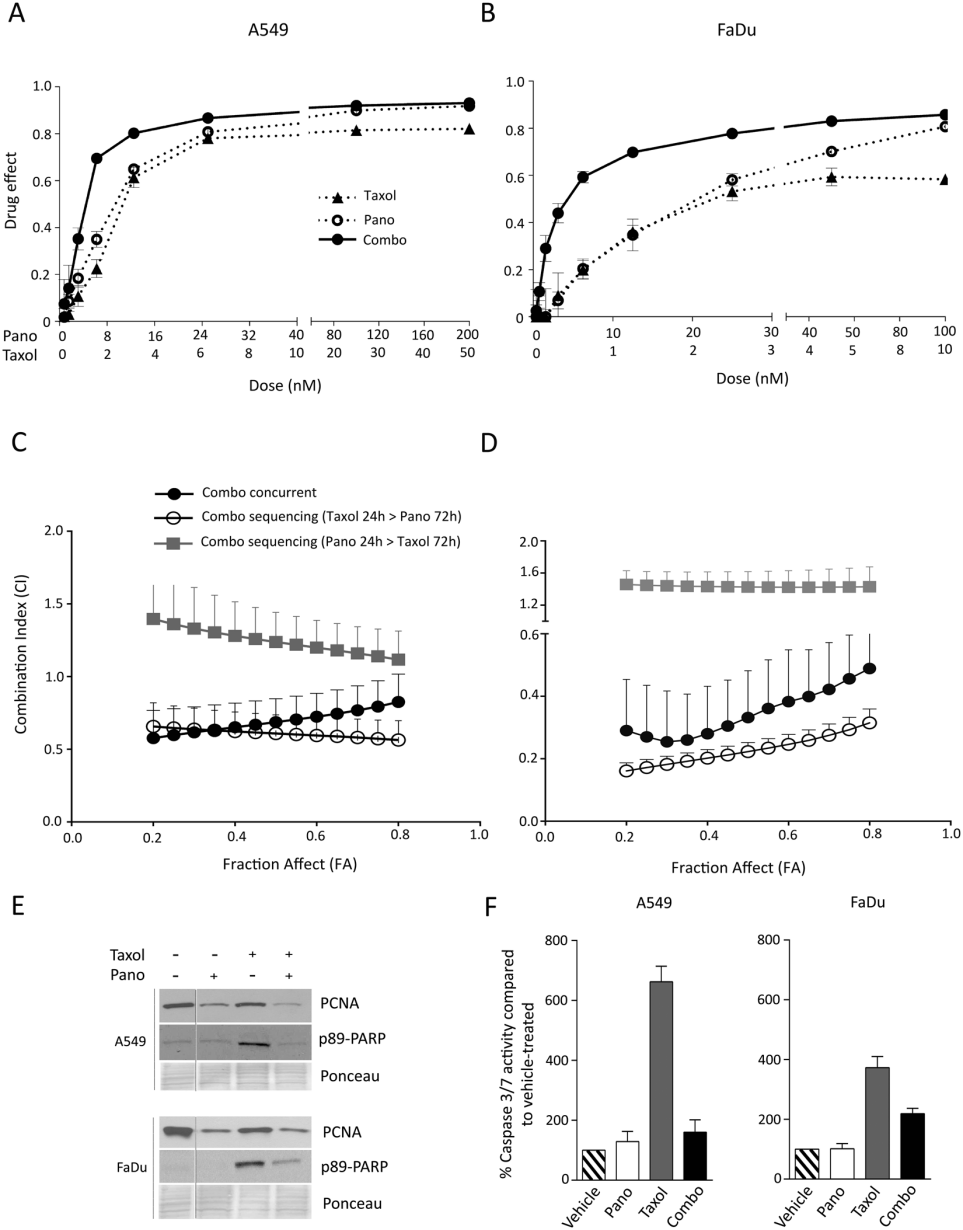
Synergy Between Taxol and Pano is Associated with Suppression of Proliferation in NSCLC and HNSCC. Dose response curves for Taxol, or pano and the concurrent combination in (**A**) A549 (NSCLC) and (**B**) FaDu (HNSCC) cells. Combination index (CI) versus Fraction Affect (FA) plots comparing concurrent and sequencing regimens, in (**C**) A549 and (**D**) FaDu. Each data point represents mean ± SEM (*n* = 6). (**E**) Immunoblot analysis for proliferation (PCNA) and cell death (p89-PARP) to evaluate the effect of low dose, concurrent Taxol and pano treatment. Doses used were: Taxol - 6.25 nM (A549), 2.5 nM (FaDu) and pano- 25 nM (both cell lines) for 72 hrs. (**F**) Caspase 3/7 activity using the same conditions outlined in **E**. Activity is indicated as % relative to vehicle treatment. Each bar represents mean ± SEM (*n* = 3).

Dose-response curves for the concurrent combination of Taxol and pano indicate a shift to the left (consistent with either additivity or synergy) in both A549 (NSCLC) and FaDu (HNSCC) cell lines (Fig. 1A and B). Subsequent analysis using the Combination Index (CI) method of Chou and Talalay^11^, an algorithm that predicts the nature and potency of drug combinations, indicate synergy in both A549 and FaDu cells (Fig. 1C and D). Similar data were obtained for other NSCLC and HNSCC cell lines (Table 1). We further evaluated the effect of drug sequencing on the interaction of Taxol and pano and determined that synergy was schedule-dependent in A549 and FaDu cells. Specifically, Taxol given prior to pano for 24 h was superior to the reverse sequence that caused pharmacologic antagonism (Fig. 1C and D). Mechanistically, pano is thought to function by inducing a G2 block^12^, which may provide an explanation for the observed antagonism when pano is given prior to Taxol.

**Table 1:** Concurrent treatment of Taxol and Panobinostat is Synergistic in Human HNSCC and NSCLC cell lines.

Cell line	IC_50_ Panobinostat (nM)	IC_50_ Taxol (nM)	Mean CI (±SD): FA = 0.2–0.8
HNSCC			
UMSCC1	19.1	4.0	0.61 (±0.33)
UMSCC47	16.6	3.8	0.70 (±0.13)
FaDu	23.6	3.8	0.37 (±0.11)
NSCLC			
A549	14.3	5.8	0.81 (±0.13)
H460	34.6	6.7	1.04 (±0.02)
H1355	31.2	17.3	0.69 (±0.30)

IC_50_ doses were calculated for pano and Taxol. Drug interactions, computed by the software Calcusyn, were expressed as a Combination Index (CI). Drug interactions were defined as: synergy = CI ≤ 1.0, additivity = 1.0, and antagonism as CI ≥ 1. Data were expressed as mean Combination Index (±SD), determined for a range of drug concentrations over a Fractional Effect (FA) of 0.2–0.8.

The synergy observed at low doses of combined drugs was associated with suppression of proliferation, evident as decreased expression of PCNA (Fig. 1E). Potentiation of cell death was not apparent in the combination treatment, as evidenced by p89-PARP immunblotting (Fig. 1E), or caspase activity (Fig. 1F), although Taxol alone demonstrated some cell kill activity.

### The Concurrent Combination of Taxol and Pano Induces Senescence

**Figure 2:**
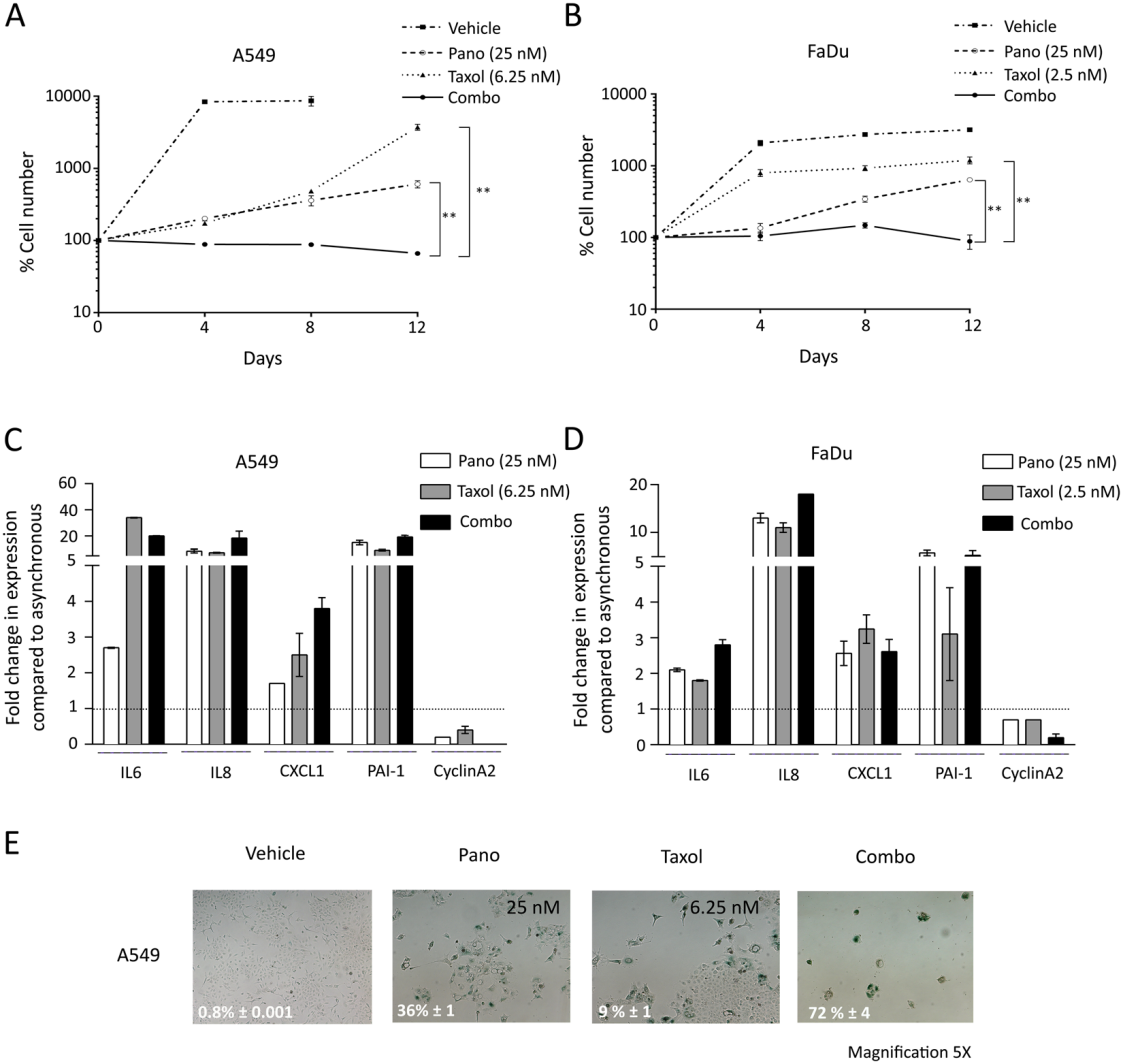
The Combination of Taxol and Pano is Synergistic due to Senescence Induction. Representative proliferation assays for (**A**) A549 and (**B**) FaDu showing sustained proliferative arrest in combination-treated cells. Cells treated with single agents exhibited transient proliferative arrest. The statistical significance (unpaired *t*-test) for combination-treated cells relative to single agents on day 12 is indicated. Transcriptome analysis (qRT-PCR) of SASP-associated genes (IL6, IL8, CXCL1, PAI-1) and proliferation (cyclin A2) in 6 day drug-treated (**C**) A549 and (**D**) FaDu cells. Increased SASP expression and absence of proliferation are typical of senescence. Bar represents mean ± SD (*n* = 3). (**E**) Representative images of SA-β-gal-stained A549 cells after 6 days treatment with the indicated doses of Taxol, pano or combo. SA-β-gal quantitation (bottom left) are indicated (mean ± SD). Magnification 5X.

Chemotherapy can result in an array of cell fates, including cell death; however the time for these fates to emerge is often delayed. Thus, the observation time following treatment was extended to clarify the long-term effect of concurrent pano and Taxol treatment. Cells treated with Taxol, pano and their combination were assayed for proliferation over 12 days to evaluate the long-term effects on cell fate. Single agents induced proliferative arrest in both cell lines, confirming the immunoblotting data shown in Fig. 1E. Analysis comparing the effect of either Taxol, or pano on proliferation at day 8 indicated statistically significant differences relative to vehicle-only treated cells (*P* = 0.0127 and 0.0131 for pano and Taxol, respectively in A549: and *P* day 8 < 0.0001 for both pano and Taxol in FaDu). This effect was transient however, and proliferation resumed after 4–6 days (Fig. 2A and B). In contrast, cells treated with the concurrent combination remained growth arrested for the duration of the experiment and for at least 4 weeks afterward.

Since senescence is a form of proliferative arrest distinct from quiescence, and is associated with the Senescence Associated Secretory Phenotype (SASP), we evaluated SASP transcription coupled with senescence-associated β-gal (SA-β-gal) activity, another commonly used assay for detecting senescence. Cells treated with single agents or the combination for 6 days had increased mRNA expression of SASP-related factors, coupled with decreased expression of CyclinA2 indicating a transcriptional signature consistent with senescence (Fig. 2C and D). Furthermore, almost all combination-treated A549 cells exhibited a flattened, granular morphology coupled with SA-β-gal positive staining (Fig. 2E). Single agents induced varying degrees of SA- β-gal positivity in localized regions, consistent with the transient growth arrest observed for single agents (Fig. 2A). The SA-β-gal assay was not informative for FaDu cells under these conditions. Thus, the concurrent combination of Taxol and pano causes prolonged proliferative arrest that is due to a high frequency of a stable senescent phenotype.

### Senescence is a Common Outcome of Cisplatin or Taxol Treatment in NSCLC and HNSCC

**Figure 3:**
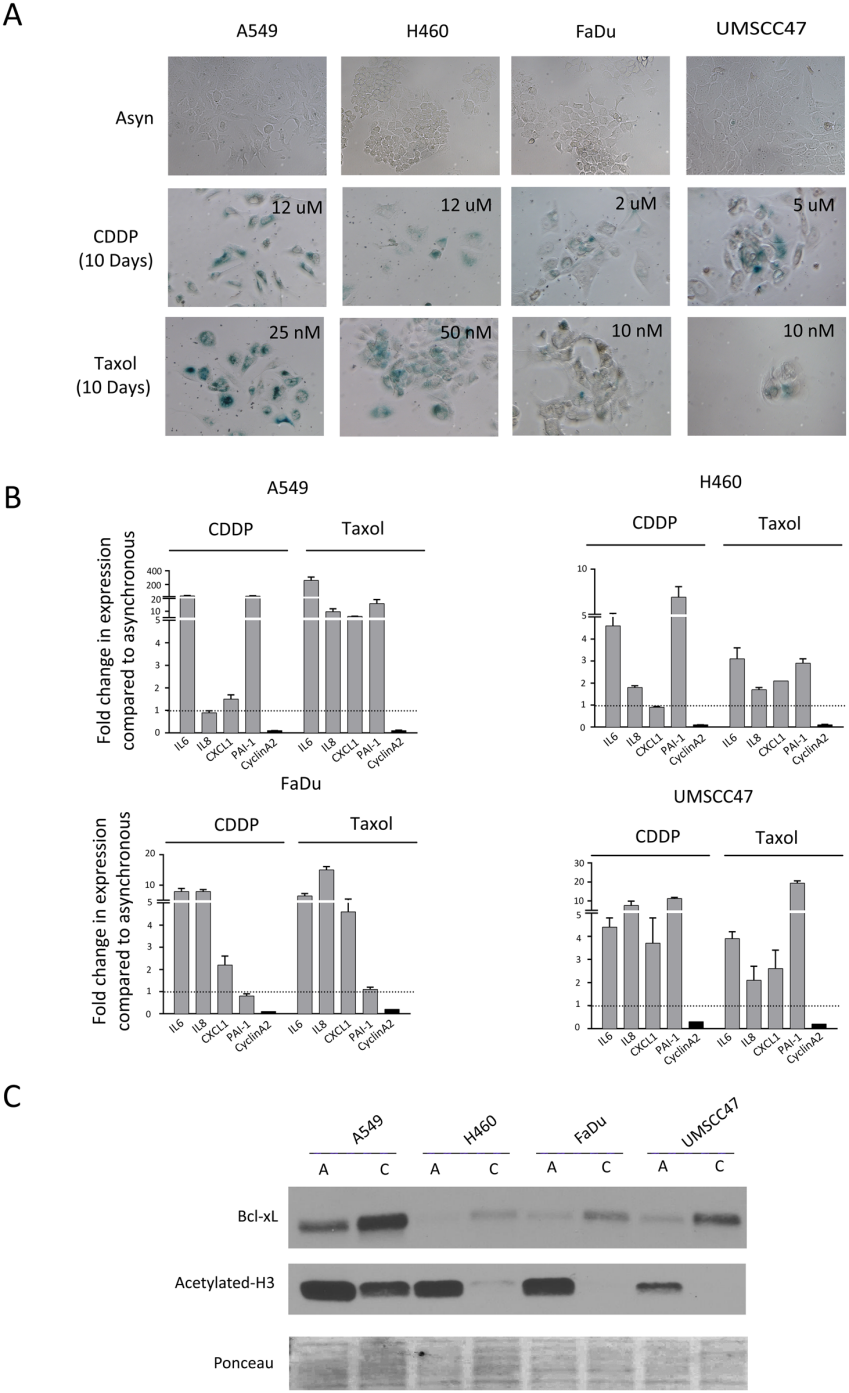
Senescence is a Common Outcome of Chemotherapy in NSCLC and HNSCC and is Associated with Altered Histone Acetylation and Bcl-xL Expression. Chemotherapy-Induced Senescent (CIS) populations were obtained by treating NSCLC (A549, H460) and HNSCC (UMSCC47, FaDu) cells with either cisplatin (CDDP) or Taxol for 10 days. The doses indicated were chosen to induce maximal cell death and residual, surviving cells typically become senescent. (**A**) Representative phase images of SA-β-gal-stained CIS populations (Magnification 10X), (**B**) Transcriptome analysis (qRT-PCR) of SASP-related gene expression and proliferation in 10 day old CIS populations. The dotted line represents baseline expression in vehicle-only treated asynchronous cells. Bars represent mean ± SD (*n* = 3) (**C**) Representative immunoblot showing decreased H3 acetylation and increased expression of Bcl-xL in CIS populations [C] compared to asynchronous [A] NSCLC and HNSCC cells. Cisplatin (CDDP) was used as the senescent inducer and doses are denoted in 3A.

NSCLC and HNSCC cells were treated for 10 days with concentrations of cisplatin (CDDP) and Taxol typically associated with initial plasma concentrations in patients. We have established that these doses cause maximal tumor cell death. The extended treatment time was critical to eliminate cells undergoing delayed death and therefore to enrich for surviving residual cells. These surviving populations were enriched for senescent cells, as measured by SA-β-gal positivity and characteristic morphology (Fig. 3A and Fig. S1), and transactivation of SASP-related factors (Fig. 3B). Although the response was heterogeneous between cell lines and drug treatments, senescent cells were present throughout. Thus, chemotherapy-induced senescence (CIS) is a common outcome in NSCLC and HNSCC cell lines.

### Characterizing CIS Populations

Senescence is a tumor suppressive mechanism^13^; however, in aging and certain diseases senescent cells accumulate^14^ due to impaired immune-mediated clearance. These findings have led to debate about the potential risks of senescent-enriched tumor populations and the elevated and prolonged inflammatory signaling that ensues^15^. Research focused on eliminating persistent senescent cells is an area of intense focus in aging and cancer therapeutics. This strategy has shown promise in transgenic mouse model studies^16^, and using therapeutic interventions that induce senescent cell death, termed “Senolytics”^17, 18^. Several drugs have been repurposed as senolytics, and the Bcl-2 family of proteins that are often overexpressed in senescent cells, are considered valid senolytic targets^18, 19^. We evaluated the expression of Bcl-2 and Bcl-xL in CIS and found increased expression of Bcl-xL in all cell lines, suggesting an association with senescence survival (Fig. 3C). Bcl-2 was not expressed in these cells.

Senescence has been shown to be associated with decreased global histone acetylation^20^. Pano treatment has been shown to increase Histone 3 (H3) acetylation^21^ and has been used as a pharmacodynamic biomarker for HDACi’s in clinical trial^22^; therefore, we evaluated whether CIS populations had altered H3 acetylation. Decreased acetylation was observed across all CIS populations evaluated (Fig. 3C). HDACi’s have also been shown to modulate the expression of Bcl-2 family proteins including Bcl-2 and Bcl-xL^23–25^, therefore we evaluated pano as a senolytic therapy to eliminate CIS, and monitored acetylated H3 in these populations as a surrogate for drug effect.

### Pano has Senolytic Activity in CIS and is More Efficacious than Repeat Dosing of Chemotherapy

**Figure 4:**
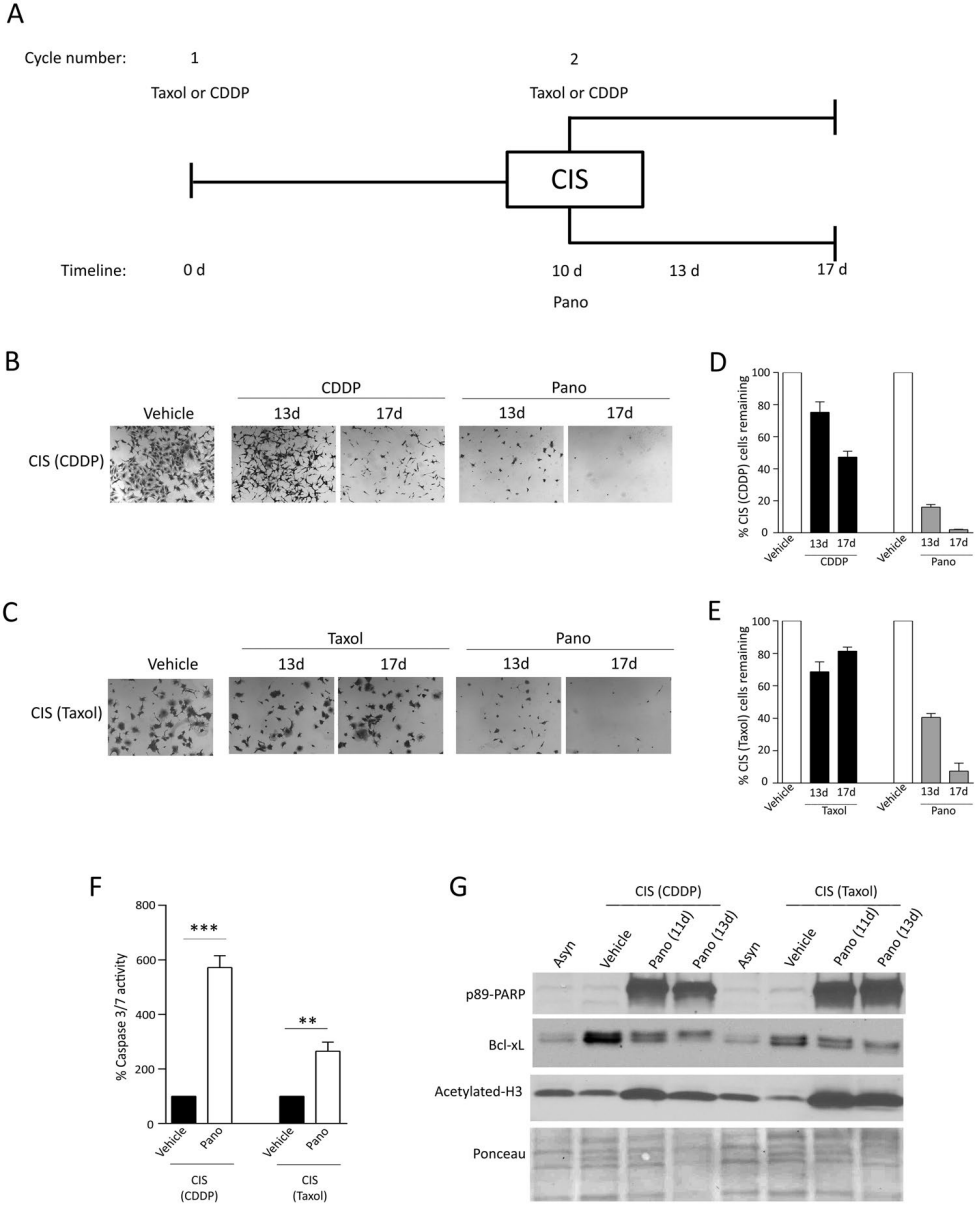
Pano has Senolytic Activity in Chemotherapy-Induced Senescent NSCLC. (**A**) Schema of experimental design. CIS populations of A549 obtained after treatment with either CDDP or Taxol (cycle 1), were subsequently treated with either (i) cycle 2 of chemotherapy (at the same dose as cycle 1), or (ii) pano (200 nM). Cells were assayed for survival at 13 and 17 days post-treatment initiation, corresponding to 3 and 7 days post-cycle 2. Representative SRB-stained images indicate dramatic cell loss following pano-treatment in (**B**) CIS (CDDP - cisplatin), or (**C**) CIS (Taxol) populations (Magnification 5X). Quantitative analysis of cell loss in (**D**) CIS (CDDP) and (**E**) CIS (Taxol) senescent populations following cycle 2 treatment (mean ± SEM, *n* = 6). Data indicate significant loss (*P* < 0.0001: unpaired *t*-test) for pano-treated cells versus chemotherapy (either CDDP or Taxol) by day 17. Vehicle controls reflect treatment of CIS populations for either duration. (**F**) Increased caspase 3/7 activity in pano-treated (3 days) CIS populations of A549. Bar represents mean ± SEM (*n* = 3) and statistical significance (unpaired *t*-test) is indicated. (**G**) Representative immunoblot showing increased PARP cleavage, decreased Bcl-xL and increased acetylated H3 in both CIS populations of A549 cells following 1- and 3-day treatment with pano (200 nM).

NSCLC and HNSCC patients are treated with repeated cycles of chemotherapy as standard of care; however, the effect of repeated doses of chemotherapy on tumor cell death, whether in proliferating or non-proliferating (senescent) cells, is largely unknown. Thus, we evaluated the ability of a 2^nd^ dose of chemotherapy to kill residual CIS populations that become enriched after a first cycle of chemotherapy. In contrast, we also evaluated pano as an intervention therapy with potential to kill senescent cancer cells when given instead of a 2nd cycle of chemotherapy. The schema is summarized in Fig. 4A. Since both CDDP and Taxol are used as first-line treatment in NSCLC, senescent cell populations derived from either drug were used. Optimization experiments indicated a dose-dependent effect of pano in CIS populations (Fig. S2). Based on these data 200 nM pano was chosen as the optimal dose to test senolytic activity.

Pano treatment resulted in dramatic cell loss in CIS populations, as shown by the representative images in Fig. 4B and C. Quantitation indicated a significant decrease in the number of adherent senescent cells remaining, as shown in Fig. 4D and E. Pano-mediated cell loss was time-dependent (Fig. 4B–E) and importantly significantly outperformed either CDDP or Taxol retreatment (unpaired *t*-test, P < 0.001)(Fig. 4D and E). Of note, CDDP-derived CIS populations were sensitive to retreatment with a 2nd cycle of CDDP, while Taxol-derived CIS populations were largely resistant to retreatment with Taxol (Fig. 4B–E). Thus, we conclude that pano has high senolytic activity.

Pano-mediated cell loss was associated with increased caspase 3/7 activity (Fig. 4F) and p89-PARP cleavage (Fig. 4G) confirming cell death, and also with decreased expression of Bcl-xL. This was more evident after 3 days in both CIS populations (Fig. 4G). Increased H3 acetylation was also observed following pano treatment, reversing the decreased trend observed in CIS.

### Pano Treatment Shows Senolytic Activity in Other CIS NSCLC and HNSCC Cells

**Figure 5:**
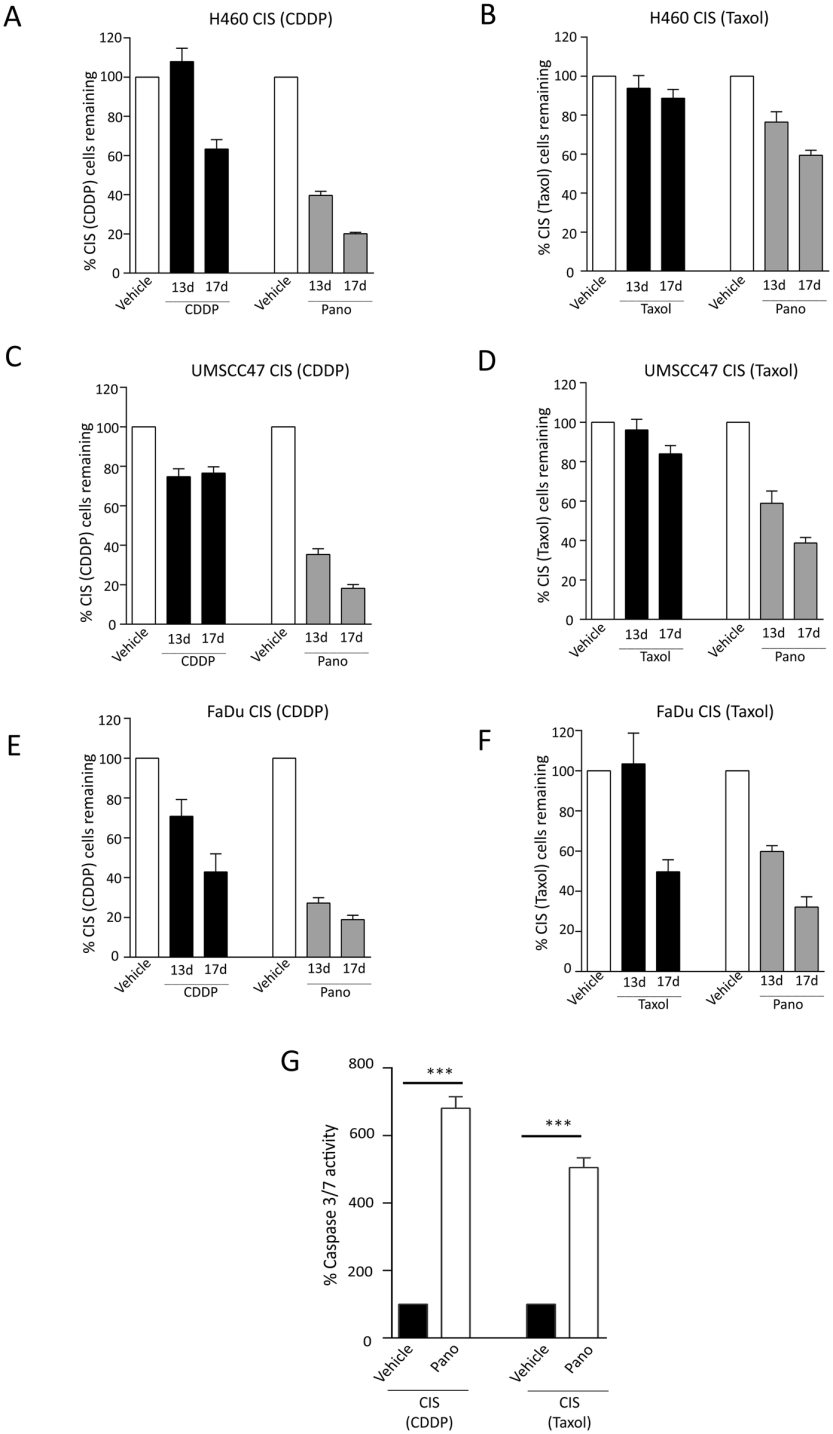
Pano has Senolytic Activity in Other Chemotherapy-Induced Senescent NSCLC and HNSCC Cells. Quantitative analysis of SRB-stained adherent CIS cells at 13 and 17 days post treatment indicating significant pano-mediated cell loss, relative to chemotherapy, in (**A**) H460 CIS (CDDP) (**B**) H460 CIS (Taxol) (**C**) UMSCC47 CIS (CDDP) (**D**) UMSCC47 CIS (Taxol) (**E**) FaDu CIS (CDDP) and (**F**) FaDu CIS (Taxol) cells. The same experimental design outlined in schema Fig. 4A was utilized. Bar represents mean ± SEM (*n* = 6). An unpaired *t*-test of pano versus chemotherapy at day 17 indicate a statistically significant difference in (**A–D**) (*P* < 0.0001); and (*P* < 0.001) in FaDu, (**E** and **F**). (**G**) Increased caspase 3/7 activity in pano-treated (3 days) CIS populations of H460 cells. Bar represents mean ± SEM (*n* = 3). *P* = <0.05: unpaired *t*-test of pano versus vehicle for both CIS (CDDP and Taxol populations).

We further evaluated the effect of pano on CIS populations derived from one additional NSCLC (H460) and two HNSCC cell lines (UMSCC and FaDu). Consistent with the findings reported for A549, pano treatment significantly outperformed either CDDP or Taxol retreatment (Fig. 5A–F). Similarly, CDDP-derived CIS populations were somewhat sensitive to retreatment with a 2nd cycle of CDDP (Fig. 5A,C and E), while Taxol-derived CIS populations were largely resistant to repeat dosing with Taxol (Fig. 5B,D and F). Pano-induced loss in CIS H460 NSCLC cells was associated with increased caspase 3/7 activity (Fig. 5G) indicating cell death; however the assay was not informative for UMSCC47 or FaDu cells, although cell detachment was evident. Immunoblotting of pano-treated UMSCC47 CIS populations did confirm PARP cleavage (Fig. S3). Similiar to A549 NSCLC, pano-mediated senolytic activity in UMSCC47 was associated with decreased Bcl-xL expression and increased H3 acetylation, indicating reproducible reversal of expression patterns observed in CIS. Thus, both Bcl-xL expression andH3 acetylation may be explored further as pharmacodynamic biomarkers of pano senolytic activity in CIS.

## Discussion

While other reports of synergy between HDACi’s and taxanes have been described^26–29^, this is the first study to determine that the mechanistic basis underlying this interaction is senescence and not potentiation of cell death. We further show that senescence is a major fate in NSCLC and HNSCC cancer cells that survive CDDP or Taxol therapy.

Although senescence limits cancer development and promotes tissue repair in young organisms, accumulation of senescent cells in aged tissue imparts risk for several reasons. Senescent cells can revert and form genomically defective daughter cells that have the potential to subsequently evolve into dysplastic cells^30, 31^. Additionally, persistent SASP secretions from senescent populations may influence autocrine and paracrine signaling within the tumor microenvironment and negatively influence disease progression^32^ and immune surveillance^33, 34^. Therefore, research efforts have recently focused on identifying and characterizing senolytic drugs for use in aging-related pathologies with obvious applications to cancer therapeutics^18^. We demonstrate here that pano can be repurposed as a senolytic drug that kills persistent senescent cancer cells that arise post-Taxol or -CDDP therapy.

Cellular targets of interest for senolytic drug development include ephrins (EFNB1 or 3), PI3KCD, p21, BCL-xL, and plasminogen-activated inhibitor-2^18^. Drugs that specifically target the BH3 domain of Bcl-2 family proteins have validated senolytic activity in non-transformed cells that have undergone replicative senescence^19^. Consistent with these findings, we observed increased Bcl-xL expression in CIS cells that was reversed upon treatment with pano, and was associated with cell death. Other reports have documented HDACi-mediated reduction of Bcl-xL expression^23, 25, 35^ shown to be due to histone deacetylation close to Bcl-xL transcription start sites, resulting in inhibition of transcription^23^. Similar to other reports^20, 36^ we also show that CIS populations have reduced histone acetylation (using H3 as a biomarker), and as expected, pano reversed this phenomenon. Aside from the effect of pano on Bcl-xL specifically, it is plausible that pano modulates the expression of other genes critical for the survival of senescent cells, although this hypothesis remains to be tested.

HDACi monotherapy has poor clinical activity; however, in combination with cytotoxic drugs and/or epigenetic modulators, anti-tumor efficacy has been noted in patients with solid tumors, as reviewed^10^. Despite encouraging activity, hematologic and other toxicities associated with HDACi combinations remain a challenge; therefore further evaluation of novel scheduling regimens is required. A review of existing clinical data^10^ indicate that trials of HDACi-containing combinatorial regimens predominately explored continuous dosing schedules resulting in a wide range of responses, but with considerable toxicity. This continuous-type dosing of HDACi’s in clinical trial makes it challenging to decipher the true impact of sequencing, or intermittent scheduling on either clinical response rate, or toxicity. Repurposing pano as a senolytic would require short-term, sequential dosing following chemotherapy to target senescent cells within the residual tumor. Further, novel dosing strategies for pano-based therapy that exploit intermittent-type treatment should be explored. Several studies of HDACi’s that demonstrated promising clinical response^37, 38^ used valproic acid, a drug that has a favorable toxicity profile, although these observations were limited to breast cancer patients suggesting a potential pharmacogenomic association.

These studies bring attention to two important topics relevant to cancer treatment. Firstly, it is largely assumed that cytotoxic drugs primarily target proliferating cells, although this dogma has been called into question^39^ since many slow proliferating tumors have an excellent response to chemotherapy; known as the ‘proliferation rate paradox’^40^. One hypothesis to account for this observation is that some cytotoxic drugs can target growth-arrested cancer cells, such as quiescent or senescent phenotypes. Indeed we demonstrate that CDDP has a cytotoxic effect on senescent cancer cells while Taxol did not. Thus it is conceivable that some cytotoxic drugs target senescent cells and thereby possess intrinsic senolytic activity. This may partially account for the clinical activity and ‘success’ of some cytotoxic agents. While this is a noteworthy observation, the senolytic efficacy of pano is notably superior to CDDP.

Secondly, there needs to be a deeper appreciation that senescence is a clinically relevant outcome of therapy. It is unclear at present if immune cell effectors can clear senescence cancer cells that arise following chemotherapy; therefore senescence should be regarded as a component of residual disease and thus, a clinically relevant contributor to therapeutic resistance. Identifying and quantifying senescence in human tumors is technically challenging and there are few published studies^41, 42^ due to a lack of senescence-specific biomarkers amenable to analysis using fresh or formalin-fixed tissue. Until this problem is adequately resolved, quantitation of ‘senescence tumor burden’ and subsequent correlation with survival cannot be thoroughly investigated. The ideal scenario for the rational evaluation of pano and other senolytic drugs should involve determination of pre- and post-chemotherapy senescence tumor burden that can be used to select patients most likely to benefit most from a post-chemotherapy senolytic intervention.

## Materials and Methods

### Drug Interaction Studies

Triplicate sets of 96-well plates were set up for treatment with Taxol, or pano alone and the concurrent combination where both drugs were combined at their equipotent molar ratio (a fixed ratio of the respective IC_50_). Six replicates were used for each drug dose evaluated, and 9 dose levels evaluated. See supplemental methods regarding experimental detail for the generation of dose-response curves. After incubation for 3 cell doublings (3–5 days), plates were processed using Sulforhodamine B (SRB)^43^, and data were input into the software Calcusyn (Biosoft, Cambridge, UK) to compute the nature of drug interaction using the method of Chou and Talalay^11^. Data were expressed as a combination index (CI) ratio; whereby CI < 1 indicate synergy, CI = 1 is additivity, and CI > 1 indicate antagonism. For drug sequencing experiments, plates were set up in the same way, but for combination treatments, drugs were added sequentially (by exposing to one drug for 24 h, aspirated and replaced with the other drug for an additional 72 h). Taxol and pano-alone controls were also modified for these experiments, whereby single agents were administered for 24 h or 72 h only. All experiments were repeated at least 3 x.

### Cell Proliferation Assays

A549 and FaDu cells were plated in 96 well plates and treated the following day with low doses of Taxol (6.25 nM for A549 and 2.5 nM for FaDu), pano (25 nM for both cell lines), or the relative combination of both. Cell numbers were counted every 4 days until day 12, using a hemocytometer. Data were plotted relative to the initial number of cells seeded. Experiments were repeated three times.

### Immunoblot analysis

Adherent and non-adherent cells were collected from drug-treated dishes and total protein extracted by Tris-SDS lysis buffer, quantified by Lowry and used for immunoblotting. Antibodies used in the study were rabbit anti-Bcl-xL (1:1000, #2764; Cell Signaling), mouse anti-Bcl-2 (1:500, Dako; #M0887), mouse anti-PCNA (1:2000,#MS106-PO; Neomarker), rabbit anti-p89-PARP (1:1000, #9541; Cell Signaling) and rabbit-anti-Acetyl-H3 (1:1000, #06-599; Millipore). Equal protein loading between lanes was confirmed by staining membranes with Ponceau S prior to immunoblotting.

### Detection of Senescence

The senescence phenotype was evaluated using multiple assays including the senescence associated β-galactosidase assay^44^; and mRNA expression of senescence-associated secretory (SASP) factors (IL6, CXCL1, IL8 and PAI-1), and cyclin A for proliferation.

### Analysis of Cell Survival Following Pano or Cycle 2 Chemotherapy

CDDP- or Taxol-derived CIS populations (10 day old) were washed 2x with warm media to remove debris and subsequently treated with (i) vehicle, (ii) the same dose of cytotoxic drug used to generate each CIS populations (see supplemental methods), or (iii) pano (200 nM). Three and seven days later monolayers were washed with warm media, and any remaining cells stained by SRB. The SRB absorbance values for three-and seven-day drug treatments were calculated (mean ± SEM) of six replicates for each condition. Experiments were repeated a minimum of 3x.

## Electronic supplementary material

Supplementary Information
