# Supplementary material for: A Novel Indication for Panobinostat as a Senolytic Drug in NSCLC and HNSCC
Source: Sci Rep. 2017 May 15;7:1900. doi: 10.1038/s41598-017-01964-1 (PMC5432488; doi:10.1038/s41598-017-01964-1)
Supplement: Supplementary file 1 — Supplementary Information [file 41598_2017_1964_MOESM1_ESM.pdf]

A Novel Indication for Panobinostat as a Senolytic in NSCLC and HNSCC

Leleesha Samaraweera[1], Alfred Adomako[2], Alicia Rodriguez-Gabin[1] and Hayley M. McDaid*[1, 3]

[1]Department of Molecular Pharmacology, [2]Pathology, and [3]Medicine (Oncology), Albert Einstein College of Medicine, Bronx, NY 10461

***Corresponding Author:** Hayley McDaid (E-mail: hayley.mcdaid@einstein.yu.edu)

Supplemental Methods

Cell Lines and Drugs. Cell lines used were FaDu, A549, H1355 and H460 (American Type Culture Collection) and UMSCC47, UMSCC1 (Milipore), which were grown according to vendor instructions in RPMI containing 10% FBS (Life Technologies, Grand Island, NY). Taxol was obtained from the Drug Development Branch, National Cancer Institute and formulated in 100% DMSO (Sigma). Cisplatin was purchased from Sigma and formulated in saline. Pano was obtained from Novartis, Boston, MA and formulated in D5W (5% dextrose in water).

Dose-Response Curves. Cells were seeded at 1000-4000 cells per well in 96-well plates and following overnight attachment, dosed with 2-fold dilutions of drugs and a vehicle-only control, for 3 cell doublings. Six replicates were used for each drug dose evaluated and 9 dose levels evaluated. Plates were processed using the SRB method[1] and the mean absorbance (OD), representing adherent cells following drug treatment, was used to calculate drug effect. This was computed as: $(OD_{Vehicle}-OD_{Treatment})/OD_{Vehicle}$. These values were used to generate dose-response curves using Prism (Graphpad Software Inc). Calcusyn (Cambridge Biosoft) software was used to determine the nature of drug combinations.

Senescence-Associated β-Galactosidase Activity. Monolayer cultures were fixed with 2% formaldehyde and 0.2% glutaraldehyde in PBS for 10 minutes, and subsequently incubated with X-gal at pH 6 for 12–15 h at 37 °C. Cells that were blue that also had a large, flat morphology with a granulated cytoplasm, were scored as positive.

Quantitative RT-PCR. Total RNA was extracted from adherent drug-treated cells and vehicle-only control cells using RNeasy kit (Qiagen), reverse transcribed using the Vilo cDNA synthesis

kit (Life technologies) and the cDNA used in quantitative real-time PCR (qRT-PCR) to determine the expression of genes (IL8, IL6, CXCL1, PAI-1, CyclinA2 and CypB). Gene expression was normalized to CypB and expressed as fold change relative to vehicle-treated controls. Primer sequences were obtained from primer bank (http://pga.mgh.harvard.edu/primerbank/) and synthesized by Life Technologies. Each experiment was repeated at least two times, in triplicate.

Generation of CDDP- or Taxol-Derived CIS populations. NSCLC and HNSCC cells were treated with CDDP or Taxol at doses that induce maximal cell kill, for 10 days. Doses used for each cell line were:

A549s - 12 μM CDDP and 25 nM Taxol,

H460 - 12 μM CDDP and 50 nM Taxol,

FaDu - 2 μM CDDPand 10 nM Taxol,

UMSCC47 - 5μM CDDP and 10 nM Taxol.

CIS was defined as populations of cells that were enriched for senescence, according to the metrics described.

Caspase 3/7 Assay. Cells were treated with single drugs or concurrent combinations at the doses and times indicated, and assayed for caspase 3/7 activity using APO-ONE 3/7 kit (Promega, Madison, WI), according to the manufacturer's protocol. The fluorescence values were expressed as % relative to vehicle-only controls. Experiments were repeated in triplicate, at least three times.

Statistical Analysis. Graphpad Prism was used to perform unpaired students t-test.

1 Vichai, V. & Kirtikara, K. Sulforhodamine B colorimetric assay for cytotoxicity screening. *Nat Protoc* **1**, 1112-1116 (2006).

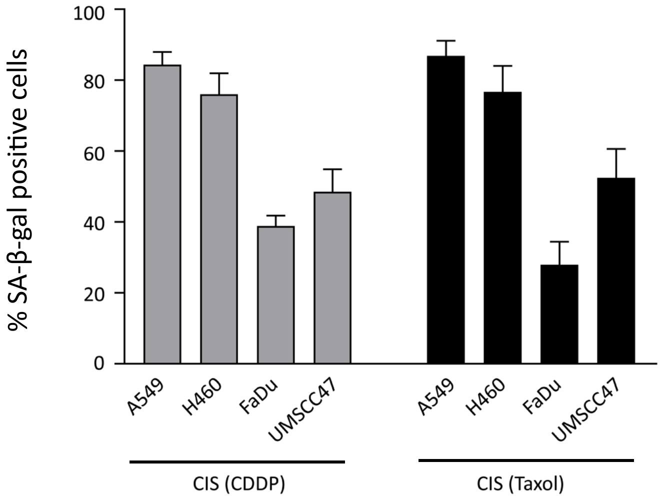

Fig. S1. Percentage of SA-β-gal Positive Cells in CIS Populations. Cells were treated with CDDP or Taxol for 10 days at doses indicated in methods, and residual cells were stained for SA-β-gal. Percent SA-β-gal positive cells (mean ± SEM) are presented (n=3).

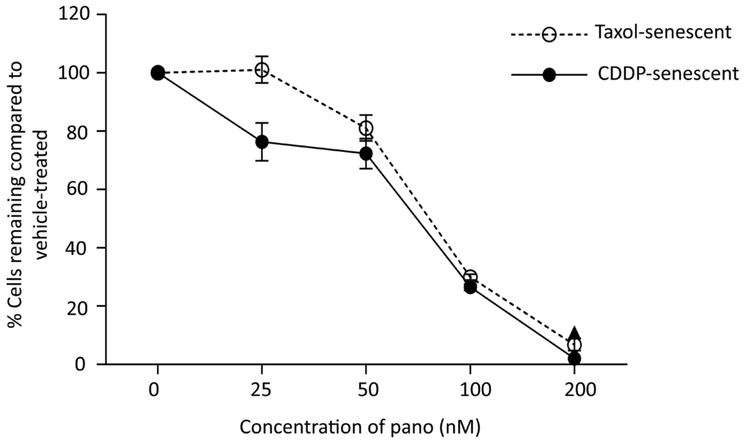

Fig. S2. Pano Treatment Causes Concentration-Dependent Cell Loss in CIS Populations. A549 cells were treated with CDDP or Taxol for 10 days (doses indicated in methods) to generate CIS populations. Subsequently, these were treated with the indicated doses of pano for 7d and processed by SRB to quantify the remaining attached cells. Each data point represents mean ± SEM (*n*=6).

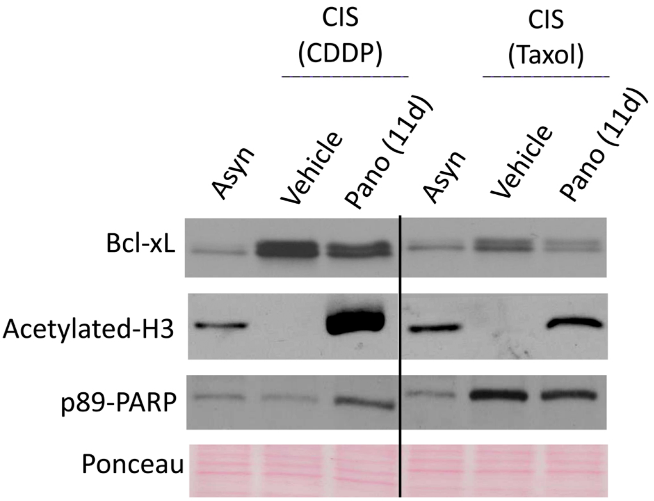

Fig. S3. Pano-mediated Senolytic Activity is Associated with Alterations of Bcl-xL and Acetylated H3 in CIS Populations of UMSCC47 Cells. Representative immunoblot showing increased p89-PARP, decreased Bcl-xL and increased acetylated-H3 following pano treatment.
